# Sex-Specific Accumulated Oxygen Deficit During Short- and Middle-Distance Swimming Performance in Competitive Youth Athletes

**DOI:** 10.1186/s40798-023-00594-4

**Published:** 2023-06-25

**Authors:** Danilo Alexandre Massini, Tiago André Freire Almeida, Anderson Geremias Macedo, Mário Cunha Espada, Joana Francisca Reis, Francisco José Bessone Alves, Ricardo Jorge Pinto Fernandes, Dalton Müller Pessôa Filho

**Affiliations:** 1 Postgraduate Programme in Human Development and Technologies, São Paulo State University (UNESP), Rio Claro, SP Brazil; 2 Department of Physical Education, School of Science (FC), São Paulo State University (UNESP), Bauru, SP Brazil; 3 CIPER, Faculdade de Motricidade Humana, Universidade de Lisboa, Cruz Quebrada, Portugal; 4 Escola Superior de Educação, Instituto Politécnico de Setúbal, Setúbal, Portugal; 5 Life Quality Research Centre, (LQRC—CIEQV, Leiria), Rio Maior, Portugal; 6 Faculdade de Motricidade Humana, Universidade de Lisboa, Lisboa, Portugal; 7 Centre of Research, Education, Innovation and Intervention in Sport and Porto Biomechanics Laboratory, Faculty of Sport, University of Porto, Porto, Portugal

**Keywords:** Pulmonary oxygen uptake, Oxygen deficit, Lean body mass, Swimming performance, Sex

## Abstract

**Introduction:**

Since sex-specific accumulated oxygen deficit (AOD) during high-intensity swimming remains unstudied, this study aimed to assess AOD during 50, 100, and 200 m front-crawl performances to compare the responses between sexes and analyse the effect of lean body mass (LBM).

**Methods:**

Twenty swimmers (16.2 ± 2.8 years, 61.6 ± 7.8 kg, and 48.8 ± 11.2 kg LBM—50% males) performed 50, 100, and 200 m to determine accumulated oxygen uptake (V̇O_2Ac_). The swimmers also performed an incremental test from which five submaximal steps were selected to estimate the oxygen demand (V̇O_2demand_) from the V̇O_2_ versus velocity adjustment. V̇O_2_ was sampled using a gas analyser coupled with a respiratory snorkel. AOD was the difference between V̇O_2demand_ and V̇O_2Ac_, and LBM (i.e. lean mass not including bone mineral content) was assessed by dual-energy X-ray absorptiometry (DXA).

**Results:**

A two-way ANOVA evidenced an AOD increase with distance for both sexes: 19.7 ± 2.5 versus 24.9 ± 5.5, 29.8 ± 8.0 versus 36.5 ± 5.8, and 41.5 ± 9.4 versus 5.2 ± 11.9 ml × kg^−1^, respectively, for 50, 100, and 200 m (with highest values for females,* P* < 0.01). Inverse correlations were observed between LBM and AOD for 50, 100, and 200 m (*r* = − 0.60, − 0.38 and − 0.49, *P* < 0.05). AOD values at 10 and 30 s elapsed times in each trial decreased with distance for both sexes, with values differing when female swimmers were compared to males in the 200 m trial (at 10 s: 2.6 ± 0.6 vs. 3.4 ± 0.6; and at 30 s: 7.9 ± 1.7 vs. 10.0 ± 1.8 ml × kg^−1^, *P* < 0.05).

**Conclusion:**

LBM differences between sexes influenced AOD values during each trial, suggesting that reduced muscle mass in female swimmers plays a role on the higher AOD (i.e. anaerobic energy) demand than males while performing supramaximal trials.

**Supplementary Information:**

The online version contains supplementary material available at 10.1186/s40798-023-00594-4.

## Key Points

- AOD increasing from 50 to 200 m was higher for female than male swimmers in all trials when expressed per unit of body weight, which seemed to be an effect of the longest time to perform each trial for females, as well as to the inability of females to increase V̇O_2Ac_ contribution relative to V̇O_2demand_, unlike as observed for males.
- AOD decreased from 50 to 200 m when analysed in a common unit of time elapsed during trials, with females showing higher values than males only in 200 m.
- The observed inverse relationship between AOD and lean body mass suggests that the higher oxygen deficit for female than male swimmers while performing short- and middle-distance races might be accounted for the sex-related differences in lean mass.
- The main message is that female swimmers performed short- and middle-distances demanding higher contribution of anaerobic sources than males by comparing AOD relative to body weight, which can be partially explained by the differences in lean body mass between sexes in view of the moderate inverse association to the parameters of AOD estimate (i.e. V̇O_2demand_, V̇O_2Ac_ and slope).

## Background

The energy demand during competitive swimming lasting ≤ 60 s (e.g. 50 and 100 m) is mainly supplied by anaerobic energy sources (60–80%), while the aerobic metabolism contributes decisively at efforts > 120 s (e.g. 200 m and longer) [1–3]. Earlier researches on anaerobic requirements in swimming reported an oxygen debt and peak blood lactate concentrations ([La^−^]) attaining (respectively) 15–18 LO_2_ and 12–18 mmol × L^−1^ during high-intensity performance [4, 5]. Furthermore, the latest assessment of energetics in high-intensity swimming performance, using the accumulated oxygen deficit (AOD) or other procedures, also supported the anaerobic metabolism as the fundamental source of energy supply in short- and middle-distances events [2, 6, 7].

In fact, for front crawl performance in 100, 200, and 400 m, respectively, at 140, 127, and 108% of maximal oxygen uptake (V̇O_2max_), the AOD contribution to the total energy demand attained 45–50, 30–35 and 15–20%, respectively, with higher conditioning level swimmers exhibiting the lower values in such range, in spite of the higher absolute AOD values and faster velocities at such exercise intensities [8]. Complementarily, when swimming performance is limited to 30, 60, and 120–180 s (probably corresponding to the 50, 100, and 200 m events), the AOD contribution attained 65–70, 50–55, and 30–35% of total energy demand [9]. However, these percentages of AOD contribution seem to be higher than those estimated for the 100 and 200 m trial performances [10], in spite of the alignment with the percentage of AOD contribution reported for similar time performances in running [11, 12], as well as with the percentage of anaerobic contribution assessed during the 100 and 200 m in swimming with methods estimating the phosphagen and glycolytic responses from body weight or oxygen debt equivalencies, and blood lactate accumulation [3, 13].

Besides of the conflict between the results of AOD estimate, the eventual dissimilarities between male and female swimmers with regard to AOD response have not yet been addressed, and therefore, the lack of an AOD women-specific response in swimming has been precluding scientists and coaches to advance regarding the conditioning requirements to enhance high-intensity performance of women. The available information is limited to the swimming economy profile (Fernandes et al. [14], 15) and maximal anaerobic capacity estimated through the maximal value of AOD, with values attaining 53.3 versus 42.7 ml × kg^−1^ (respectively) for male and female swimmers at 120 and 180 s exertions (approximately 200 and 300 m; Ogita et al. [16]).

Supposedly, the difference between sexes is justified by the larger active muscle mass in males, but basic statements about sex-related differences affecting AOD values (like lean body mass) are still lacking. However, when considering that AOD is assessed by subtracting the accumulated oxygen uptake (V̇O_2Ac_) from the predicted O_2_ demand (V̇O_2demand_) in exercises performed at intensities higher than that corresponding to V̇O_2max_ (i.e. > iV̇O_2max_, Bangsbo et al. [17]), then the AOD sex-specific response at such supramaximal intensities might be supposedly an effect of the limited oxygen (O_2_) supply among those with reduced lean body mass; therefore, a reduced V̇O_2Ac_ is hypothesized for female swimmers.

This preliminary speculation is supported by the statements that females have limited capacity to deliver O_2_ to the working muscles at maximal V̇O_2_ rates [18, 19] and that blood volume and haemoglobin mass are closely related to lean body mass in young and healthy individuals [18], therefore such assumption as higher AOD values are expected for female swimmers during 50, 100, and 200 m front crawl, as a function of low lean body mass, allows us to hypothesize that lean body mass relates negatively to AOD.

In addition, another factor affecting AOD in swimming might be the effect of hydrodynamics on V̇O_2_ versus velocity slope [1, 15], since the alteration of this slope is the key factor modifying V̇O_2demand_ estimation between subjects and determining differences of AOD values in running and cycling [20]. Nevertheless, the hydrodynamic effect on V̇O_2_ versus the linear velocity relationship in swimming relates to conditioning level and not a sex-based characteristic [14, 15], and therefore, the AOD sex-specific response (when observed) probably relies on the lower V̇O_2Ac_ in female than male swimmers. Thus, to address the effect of sex on AOD in the 50, 100, and 200 m front crawl performances, the current study aimed to estimate and compare AOD between male and female swimmers at the total and common partial time elapsed (i.e. isotimes) and also, determine the relationship between AOD and lean body mass in short- and middle-distance events.

## Methods

### Subjects

Twenty swimmers (10 males and 10 females) from the Aquatic Sports Association team (Bauru, Brazil) volunteered to participate in the current study. Their main body characteristics were: 16.9 ± 2.1 versus 15.5 ± 3.1 years old, 179.5 ± 7.3 versus 161.6 ± 7.5 cm in height, 69.3 ± 8.1 versus 54.4 ± 6.8 kg of body mass, 12.2 ± 3.1 versus 23.3 ± 4.0% of body fat, and 57.9 ± 7.0 versus 39.7 ± 5.5 kg of lean body mass, respectively. The swimmers best front crawl performances at 50, 100, and 200 m achieved 582 ± 91 versus 533 ± 62, 616 ± 92 versus 530 ± 72, and 607 ± 93 versus 552 ± 93 FINA points for male and female swimmers, respectively, and their personal best time performance corresponded to 80 and 77% of the actual 200 m world junior records (in a 25 m pool) for male and female swimmers, respectively. All participants (and their legal guardians when under 18 years old) signed an informed consent. This research was approved by the local Ethics Committee of São Paulo State University (CAEE: 54372516.3.0000.5398), which therefore supported that the procedures adhered to international and national laws for ethics principles in research practice with human participants.

### Study Design

After being familiarized with the experimental procedures, and after a standard low-moderate intensity warm-up [3], the swimmers performed four testing sessions (separated by 24 h) in a 25 m indoor swimming pool (~ 28 ºC water temperature and ~ 50% relative humidity) always at the same time of day. Firstly, they swum 50, 100, and 200 m maximal front crawl trials in randomized order and afterwards performed an incremental intermittent protocol until exhaustion composed of 6 × 250 plus 1 × 200 m at 50, 60, 70, 80, 90, 95, and 100% of the velocity corresponding to the previous 200 m trial, with 30–60 s intervals between steps [21]. Participants avoided exhaustive training and caffeine/alcohol ingestion 24 h before the tests and were well fed and hydrated.

### Measurements

The 50, 100, and 200 m trials and the incremental protocol used in-water starts and open turns (without underwater gliding), with the swimming speed of the incremental protocol steps being controlled visually through an underwater LED line (Pacer2Swim®, KulzerTEC, Aveiro, Portugal), previously applied to pace control in swimming [21, 22]. Gas exchange was sampled breath-by-breath using a gas analyser (K4b^2^® Cosmed, Rome, Italy) attached to a breathing snorkel (New AquaTrainer®, Cosmed, Rome, Italy), ensuring oxygen uptake (V̇O_2_) sampling accuracy [23]. Raw V̇O_2_ data were smoothed every three breaths, aligned for time, interpolated second-to-second and averaged every 30 s [22, 24]. The peak oxygen uptake (V̇O_2peak_) was considered as the higher moving average attained during the incremental protocol and its velocity (e.g. vV̇O_2peak_) corresponded to the step in which it was attained [15, 22].

The V̇O_2_ versus velocity relationship during the incremental protocol was adjusted with a linear function (*y* = *ax* + *b,* where *y* is V̇O_2_, *x* is swimming velocity, *a* is the slope, and *b* is the intercept), a reliable procedure to estimate the V̇O_2demand_ [11]. For ensuring that each step lasted ~ 3–4 min, minimizing extra V̇O_2_ occurrence and the nonlinearity between V̇O_2_ response and velocity [20], only the five intermediate incremental protocol steps entered the model. This selection also aimed to elicit a V̇O_2_ demand close to an isocapnic condition to maintain V̇O_2_ aligned with the velocity increments [25] and a 10–35 s V̇O_2_ kinetics time constant that is typical for moderate and heavy intensity domains [24]. The final V̇O_2_ response in each step was obtained by the last 30 s moving average value [3].

The V̇O_2Ac_ values over the 50, 100, and 200 m trials were obtained by integrating V̇O_2_ versus time response for each distance using Eq. 1 [7]:

1 $${\dot{\mathrm{V}}\mathrm{O}}_{2\mathrm{Ac}}={\int }_{{t}_{0}}^{{t}_{\mathrm{Lim}}}{\dot{\mathrm{V}}\mathrm{O}}_{2}\times \mathrm{d}t -\left({\dot{VO}}_{2\mathrm{baseline}}\times {t}_{\mathrm{Lim}}\right)$$

where *t*_0_ and *t*_Lim_ refer to each front crawl trial onset and final times. AOD was estimated by subtracting V̇O_2Ac_ from V̇O_2demand_, considering 9% of O_2_ stores in blood and muscle [26]. From Eq. 1, AOD was also calculated at 10 and 30 s isotimes for each 50, 100, and 200 m trial, considering *t*_0_ → *t*_Lim10s_ and *t*_0_ → *t*_Lim30s_ to determine V̇O_2Ac_ over the first 10 and 30 s, respectively. V̇O_2demand_ was estimated by extrapolation of the linear function between V̇O_2_ and velocity to the respective velocity in 50, 100, and 200 m trials, considering V̇O_2baseline_ as a fixed intercept. The V̇O_2demand_ for each isotime was estimated by limiting V̇O_2_ projection to 10 and 30 s elapsed during the 50, 100, and 200 m trials. Blood was sampled (25 μl) during rest, at every interval of the incremental protocol (at 1, 3, 5 and 7 min of the recovery after each effort) for assessing [La^−^] values (using a YSL analyser, 2300 STAT, Yellow Springs). Dual-energy absorptiometry (DXA, Hologic®, QDR Discovery Wi®) was used to assess body composition, with APEX® software providing whole-body values for lean body mass (LBM, i.e. not including bone mineral content), and body fat percentage (%F).

### Statistical Analysis

The Shapiro–Wilk and Mauchly tests verified data normality and sphericity. An independent t-test (2-tailed) compared sexes regarding body composition, V̇O_2max_, and vV̇O_2max_, with the corresponding effect size being calculated using Hedges *g*: < 0.19 [trivial], 0.20–0.49 [small], 0.50–0.79 [medium], 0.80–1.29 [large], and > 1.30 [very large] [27]. Two-way ANOVA (with Sidak as post hoc) compared the effect of sex and exercise distances on AOD. The effect size was calculated using Partial eta squared (*η*^2^*p*), considering 0.0099 [small], 0.0588 [medium], and 0.1379 [large] [28]. The correlations between AOD, V̇O_2demand_, V̇O_2Ac_, slope, and lean mass were analysed by Pearson’s coefficient (*r*), with and without sex as a control variable, and classified as 0–0.29 [negligible], 0.3–0.49 [low], 0.5–0.69 [moderate], 0.7–0.89 [high], and 0.9–1.0 [very high] [29], with the significance level being set at *P* ≤ 0.05.

## Results

Table 1 displays the values of the variables obtained from the incremental protocol and the 50, 100, and 200 m maximal trials. The V̇O_2peak_ relative to body weight did not differ between male and female swimmers (*P* = 0.34, *g* = 0.42 [small]), although a higher vV̇O_2peak_ was observed in the males (*P* = 0.01, *g* = 1.18 [large]). During the incremental test, the variables respiratory exchange ratio (1.00 ± 0.09), peak blood lactate (8.4 ± 3.1 mmol × L^−1^), and percentage of age-predicted maximal heart rate (92 ± 6 bpm) reached all values corresponding to the maximal exertion. The values of the constants (e.g. slope and intercept) for the V̇O_2_ versus velocity adjustment during the incremental protocol were similar between sexes (*P* = 0.36, *g* = 0.44 [small]) and *P* = 0.975, *g* = 0.01 [trivial], respectively). Complementarily, this adjustment showed a high linear coefficient (*R*^2^) (0.99 ± 0.006) for the steps encompassing the longest exercise duration (258.0 ± 20.7 s) at the lowest exercise intensity (63.4 ± 8.6%V̇O_2peak_) to the shortest exercise duration (208.4 ± 15.8 s) at the highest exercise intensity (91.2 ± 6.4%V̇O_2peak_). The 50, 100, and 200 m exertions were conducted at 128.3 ± 11.6 versus 126.3 ± 9.2, 114.2 ± 6.2 versus 117.3 ± 7.5, and 98.6 ± 5.9 versus 100.4 ± 6.0% of the vV̇O_2peak_ for male and female swimmers, respectively, without differences between sexes (*P* = 0.42, *η*^2^*p* = 0.042 [small]).

**Table 1:** Mean ± SD and standard error of measure values of the main variables obtained in the front crawl incremental protocol and 50, 100, and 200 m trials

	Men (*n* = 10)			Women (*n* = 10)			Total (*n* = 20)		
	Mean ± SD	IC_95%_	SEM	Mean ± SD	IC_95%_	SEM	Mean ± SD	IC_95%_	SEM
*Step protocol*									
V̇O_2peak_ (ml × kg^−1^ × min^−1^)	58.8 ± 5.5	54.8–62.7	1.7	56.1 ± 6.7	51.1–60.9	2.1	57.4 ± 6.2	54.5–60.3	1.38
vV̇O_2peak_ (m × s^−1^)	1.29 ± 0.07*	1.24–1.34	0.02	1.21 ± 0.07*	1.17–1.25	0.02	1.25 ± 0.08	1.21–1.29	0.02
Slope (ml × kg^−1^ × m^−1^)	0.49 ± 0.08	0.43–0.55	0.03	0.54 ± 0.13	0.45–0.63	0.04	0.51 ± 0.11	0.46–0.56	0.02
Intercept (ml × kg^−1^)	12.6 ± 4.6	9.3 ± 15.9	1.45	12.6 ± 2.9	10.5 ± 14.7	0.92	12.6 ± 3.7	10.9 ± 14.4	0.82
*50 m*									
Velocity (m × s^−1^)	1.66 ± 0.17*^bc^	1.54–1.78	0.05	1.52 ± 0.08*^bc^	1.46–1.57	0.03	1.59 ± 0.15^bc^	1.52–1.66	0.03
AOD (L)	1.37 ± 0.24^bc^	1.19–1.54	0.08	1.33 ± 0.24^bc^	1.16–1.50	0.07	1.35 ± 0.24^bc^	1.24–1.46	0.05
V̇O_2Demand_ (L)	2.07 ± 0.24*^bc^	1.90–2.25	0.08	1.81 ± 0.26*^bc^	1.62–1.99	0.08	1.94 ± 0.28^bc^	1.81–2.07	0.06
V̇O_2Ac_ (L)	0.57 ± 0.15*^bc^	0.47–0.68	0.47	0.34 ± 0.11*^bc^	0.27–0.42	0.03	0.46 ± 0.17^bc^	0.38–0.54	0.04
*100 m*									
Velocity (m × s^−1^)	1.48 ± 0.12^ac^	1.39–1.56	0.04	1.41 ± 0.07^ac^	1.36–1.46	0.02	1.44 ± 0.10^ac^	1.40–1.49	0.02
AOD (L)	2.08 ± 0.67^ac^	1.60–2.55	0.21	1.97 ± 0.29^ac^	1.76–2.18	0.09	2.02 ± 0.50^ac^	1.79–2.26	0.11
V̇O_2Demand_ (L)	4.32 ± 0.54*^ac^	3.94–4.71	0.17	3.69 ± 0.49*^ac^	3.34–4.04	0.15	4.00 ± 0.57^ac^	3.73–4.28	0.13
V̇O_2Ac_ (L)	2.04 ± 0.32*^ac^	1.81–2.27	0.10	1.52 ± 0.28*^ac^	1.32–1.72	0.09	1.78 ± 0.40^ac^	1.60–1.97	0.09
*200 m*									
Velocity (m × s^−1^)	1.27 ± 0.09^ab^	1.21–1.34	0.03	1.21 ± 0.08^ab^	1.15–1.26	0.02	1.25 ± 0.09^ab^	1.20–1.28	0.02
AOD (L)	2.87 ± 0.74^ab^	2.34–3.40	0.24	2.99 ± 0.68^ab^	2.50–3.48	0.22	2.93 ± 0.70^ab^	2.60–3.26	0.17
V̇O_2Demand_ (L)	9.00 ± 1.12*^ab^	8.20–9.80	0.35	7.60 ± 1.07*^ab^	6.83–8.36	0.34	8.30 ± 1.28^ab^	7.70–8.90	0.29
V̇O_2Ac_ (L)	5.85 ± 0.60*^ab^	5.42–6.28	0.19	4.31 ± 0.74*^ab^	3.78–4.83	0.23	5.08 ± 1.03^ab^	4.60–5.56	0.23

Data are depicted by sex and for the total sample

a, b, c differences between 50, 100, and 200 m trails (respectively) and ^*^ differences between sexes (*P* ≤ 0.05)

Obs.: the coefficient of linear regression adjusted to the sample *N* (*R*^2^_adj_) corresponded to 0.99 ± 0.009, 1.00 ± 0.003, and 0.99 ± 0.007; and the Standard error of estimate (SEE) attained 0.03 ± 0.01, 0.02 ± 0.01, and 0.02 ± 0.01 ml × kg^−1^ × min^−1^, respectively, for men, women and the entire group of participants

**Fig. 1:**
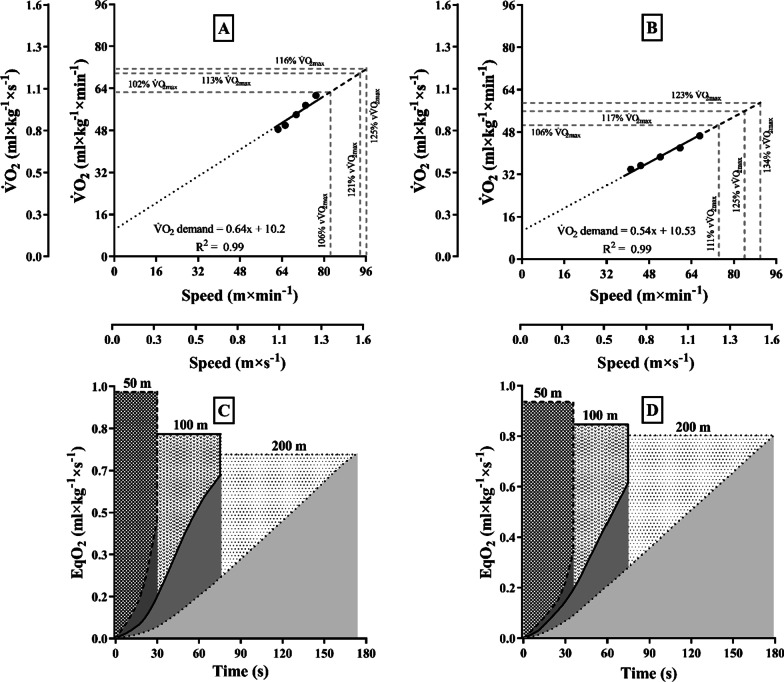
Individual example of the V̇O_2demand_ estimating (Panels A and B for male and female swimmers, respectively) and AOD profile during maximal 50, 100, and 200 m front crawl trails, with partial AOD increase being depicted at isotime 30 s for each swimming distance (Panel C and D for male and female swimmers, respectively). Obs.: In Panel **A**, the standard error of estimate (SEE) = 0.01 ml × kg^−1^ × min^−1^. In Panel **B**, the areas hachured and graded in grayscale correspond to AOD and V̇O_2Ac_ for each distance, and the line at the top of each area depicts V̇O_2Demad_ over time

**Fig. 2:**
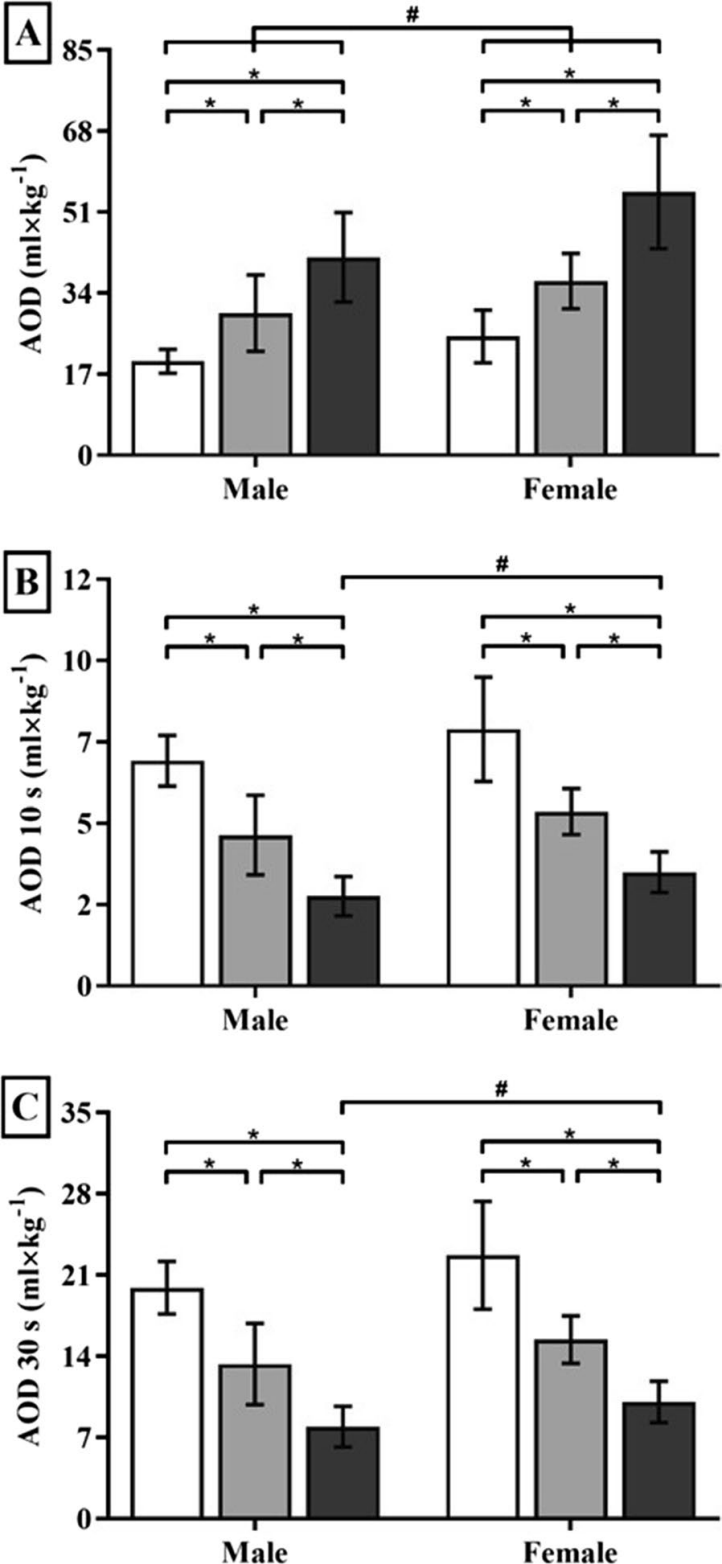
Total AOD, and partial time analysis values at 10 and 30 s isotimes, for male and female swimmers in each studied distance, with white, light grey, and dark grey bars depicting 50, 100, and 200 m trials (respectively). ^#^ and * represent differences between sexes and distances (respectively). See text for further details of statistical analysis

Figure 1 illustrates the V̇O_2demand_ estimate (in Panel A) and the AOD profile at a given period of time and for the entire distance in 50, 100, and 200 m maximal front crawl trials (in Panel B). It is observed that there is a tendency to attain high absolute AOD values (i.e. not normalized per unit of body weight) as an effect of both the intensity and duration of the performances, with no differences between sexes—i.e. AOD is greater at higher swimming speeds (50 and 100 m) due to the greater deficit at the beginning of the performance, but at long distances (200 m) there is greater accumulation with time, whatever the sex. The effect of body mass on AOD values (i.e. relative to body weight) is depicted for each trial in Fig. 2 (Panel A), revealing differences (*P* = 0.04, *η*^2^*p* = 0.190 [large]) between: (i) distances, with relative AOD values increasing from 50 to 100 m, 50 to 200 m, and 100 to 200 m in both male and female swimmers (all for *P* < 0.01); and (ii) sexes, with higher relative values for female swimmers in 50 m (*P* = 0.01), 100 m (*P* = 0.05), and 200 m (*P* = 0.01). The variability of the absolute AOD estimates for the entire sample at 50, 100, and 200 m demonstrated low heterogeneity for the group (Table 1).

Inverse relations were observed between AOD and LBM for 50, 100, and 200 m trials (*r* = − 0.60 [moderate], − 0.45 [low] and − 0.49 [low], all for *P* ≤ 0.05), but when adjusted for sex no relationship was found. Since LBM values differed between male and female swimmers (CI_95%_: 52.9–62.9 vs. 35.8–43.7 kg;* P* < 0.01), the previously observed correlation to AOD showed to be an effect of the differences in LBM between sexes.

Before controlled for sex, LBM inversely related with V̇O_2demand_ in 50, 100, and 200 m trials (*r* = − 0.56 [moderate], − 0.51 [moderate] and − 0.45 [low], *P* ≤ 0.05) and slope (*r* = − 0.44 [low], *P* = 0.03). After controlled for sex, LBM versus V̇O_2demand_ relations at each distance (*r* = − 0.51 [moderate], -0.46 [low] and -0.40 [low], respectively, for the 50, 100, and 200 m, *P* ≤ 0.05) presented small reductions, while for LBM versus slope the correlation increased (*r* = − 0.49 [low], *P* = 0.03). There were relationships between LBM and V̇O_2Ac_ only when adjusted for sex (*r* = − 0.41[low], − 0.70 [high] and − 0.53 [moderate], respectively, for the 50, 100, and 200 m, *P* ≤ 0.04).

By comparing relative AOD values at 10 s (*P* = 0.81, *η*^2^*p* = 0.011 [medium]) and 30 s (*P* = 0.82, *η*^2^*p* = 0.011 [medium]) elapsed times during the 50, 100, and 200 m trials, it was possible to illustrate the effect of exercise intensity at each distance per sex (Fig. 2 Panel B and C). Both sexes reduced relative AOD values at 10 and 30 s from 50 to 100 m, 50 to 200 m, and 100 to 200 m (with *P* < 0.01 for all comparisons). No differences between sexes were observed at 10 and 30 s for 50 and 100 m trials. However, male swimmers presented lower values than females at 10 and 30 s in the 200 m (*P* = 0.02 at both isotimes).

## Discussion

The anaerobic energy contribution in short- and middle-distance swimming is fundamental for higher level performances [8–10, 16], but its analysis per sex remains unstudied. Thus, we have determined the AOD of male and female swimmers at the 50, 100, and 200 m front crawl distances, probably some of the most relevant events of the Olympic Games. The current study reported three unique findings: (i) the absolute and relative AOD values increased from 50 to 200 m front crawl trials for both sexes, with female swimmers exhibiting higher values than males only when AOD is expressed in relative terms; (ii) the relative AOD values at 10 and 30 s elapsed times in each distance-trial decreased for both sexes (concurrently with velocity reducing as distances increased), with higher values for female swimmers than males in the 200 m trial; and (iii) LBM and relative AOD values are inversely related for all distances, probably due to the differences in body composition between sexes.

Both groups showed time dependence for the AOD profile from 50 to 200 m front crawl trials, which is aligned to previous studies reporting AOD for swimming performances at 140, 127, and 108% V̇O_2max_ [8] and for bouts lasting 30, 60, and 120 s [6, 10]. In these pioneer studies, conducted only with male swimmers, the AOD profile reaches its maximal hypothetical value of 3.2 LO_2_ when performing at 108% V̇O_2max_ [8] or either during exhaustive trials performed at 100–110% vV̇O_2max_ lasting 120–180 s [10]. In the current study, the mean absolute AOD values (in litres) for both sexes during the 200 m are close to the aforementioned maximal reference, and therefore, we evidenced that male and female swimmers only differed in AOD response when body mass differences are not taken in account.

Regarding the differences between sexes for AOD, only Ogita et al. [16] reported the maximal AOD was higher for male (53–61 ml × kg^−1^) than female swimmers (43–53 ml × kg^−1^) and stated that AOD reaches its maximum value in swimming performances lasting 2–3 min, at a metabolic rate of 110% V̇O_2peak_. Although these results for maximum AOD values between sexes are unique in swimming, they are in line with the trend of values presented in other sports [11, 30, 31]. Despite the maximal AOD assessment is not in the scope of the current study, the AOD values for male and female swimmers during 200 m correspond to an overall energy demand approaching 107% and 115% V̇O_2peak_, respectively; therefore, the current data suggest that the 200 m might be an adjustable distance to demand the maximal AOD in female swimmers, while for males a longer distance at such metabolic rate would be advisable.

However, AOD increases over time, but its contribution to total V̇O_2demand_ reduces as the period of swimming time is elongated, with values lower for male swimmers than females, respectively, in the 50 (65 vs. 73% V̇O_2demand_), 100 (47 vs. 53% V̇O_2demand_), and 200 m (32 vs. 39% V̇O_2demand_). These observed AOD rates of contribution are close to those for 30, 60, and 120–180 s reported by Ogita et al. [9] and for 30 s tethered swimming bouts reported by Peyrebrune et al. [6]. Curiously, those rates for AOD contribution are also similar to those observed in cycling during 30, 60, and 120 s (60, 50, and 35%, respectively; [32], and running the 400 (59% and 55%) and 800 m events (40 and 30%), respectively, for male and female participants [12]. Hence, these reports support the notion that the equilibrium of contribution (50–50%) between AOD and V̇O_2Ac_ (i.e. anaerobic vs. aerobic activation) in swimming is attained between 100 and 200 m, regardless of sex.

The results also showed that LBM related negatively to the relative AOD values (for each distance), V̇O_2demand_, and to the slope of V̇O_2_ versus velocity adjustment. While the LBM correlation to AOD reduced when controlled for sex-specific body composition, the V̇O_2Ac_, V̇O_2demand,_ and the slope remained unchanged, or enhanced and became significative. These correlations suggest that swimmers with the greatest LBM can perform each distance with the highest oxidative energy contribution, which is supported by the higher V̇O_2Ac_ and lower AOD contributions to V̇O_2demand_ for male swimmers when compared to females in each distance.

Studies relating muscle tissue mass to metabolic response also consider that the largest is the muscle mass engaged in exercise, the lowest is power produced per unit of muscle mass, reducing the glycolytic demand and increasing the oxidative energy supply when comparing different exercises performed at a similar percentage of V̇O_2max_ [33]. Regarding sex-specific lean mass regionalisation, larger upper-limb muscle mass provides less peripheral restriction and higher O_2_ muscle extraction, increasing V̇O_2Ac_ [31]. Therefore, the current study corroborated the influence of muscle mass on relative AOD values, considering that sex-specific response is mandatorily lower among swimmers with greater LBM while performing trials at not different %V̇O_2peak_ and %vV̇O_2peak_ during the same distance.

However, the findings that the maximal AOD values differed between males and females in running and cycling have been attributed to the specificity of sport demand and conditioning level, with reduced interference (4%) accounting for the sex difference regarding active muscle mass [30]. Nevertheless, the sex-specific amount of active muscle mass influences peak oxygen deficit in leg-cycling, demonstrating that oxygen deficit increases for both sexes when comparing one versus two-leg cycling, with higher values for men accounted to the lager fat-free leg volume [34]. Furthermore, large muscle mass engagement also supports the findings that anaerobic capacity measured by the Wingate (30 s “all-out”) test with leg and arm ergometers differed between sexes only for upper-limbs, supporting the statement that body weight does not account for sex differences, exceptionally for body regions with distinct LBM distribution [31].

For the current study, both statements suggest that differences in body weight and LBM between sexes were associated with AOD increase in 50, 100, and 200 m. However, body composition differences between sexes neither constraint the absolute AOD values nor the rate of oxygen deficit (e.g. AOD measured at fixed elapsed times) during each bout, suggesting that female swimmers have higher relative AOD demand than males while performing supramaximal trials regardless of the distance and duration of the performance.

Regarding the slope, which is a rate relating V̇O_2_ to submaximal velocities, it is an index of the oxygen cost for the increment in exercise intensity [25, 35], which accounts for more than half of the variance in the AOD estimation [26]. In the current study, the difference between sexes for the slope was about 8.8%, with female swimmers showing higher values than males. The observed difference in slope between sexes might be considered not too large, when comparing with other studies in swimming that reported slopes differing from 13 to 25% between sexes [15, 25].

In general, male swimmers possess higher slope values than females when reported in values not relative to body weight (i.e. ml × min^−1^), which is a feature of high hydrodynamic drag during higher velocity for men [15]. However, the current slope values were presented relative to body mass, and hence, best aligned to the notion that higher cost for female swimmers might suggest a premature demand upon less economical fibre types while exercise intensity increases [25], and therefore, also aligned to the increased oxygen deficit and reduced exercise tolerance at such a high-intensity condition [31]. Notwithstanding, the effect of low LBM on O_2_ availability to muscles at high-intensity exercise [18] is also aligned to the notion of the earliest recruitment of fast-type fibres, and hence, explains the inverse relationship of slope and V̇O_2Ac_ with LBM observed in the current study. In addition, the difference between sexes regarding body lean mass was also reported to have an effect on energy cost of swimming during middle-distance maximal performance [7].

A limitation could be related to the number of trials applied to the AOD assessment, which is not similar to the former AOD assessment. However, studies with similar procedures have been applied satisfactorily for submaximal steps during incremental tests for the assessment of AOD in swimming, running and kayaking [10, 12, 36]. Also, the number of trials and the exercise intensities (e.g. %V̇O_2peak_) applied in the current study are in accordance with the recommendation to avoid the nonlinearity of the slope and affect the robustness of the extrapolation of the V̇O_2demand_ [20]. The use of the New Aquatrainer® to measure the V̇O_2Ac_ might be considered another limitation to reproduce physiological demand ecologically if considering the delays of the actual swimming velocity as an effect of turning and gliding constraints [23]. However, the swimmer is enabled to stroke at a maximum rate when required, and therefore, task impairments with the New Aquatrainer® would not affect the muscle mass engagement, as well as the level of exertion while swimming [7, 21]. An additional limitation is the lack of information about the differences in anaerobic capacity (i.e. the MAOD) between the sexes, which precluded the analysis of the rate at which anaerobic metabolism was activated during each trial performance, as well as if the level of anaerobic conditioning has influence on time performance during 50, 100 and 200 m.

## Conclusion

In conclusion, male and female swimmers performed 50, 100, and 200 m with similar relative pacing above maximal aerobic velocity, but required different relative AOD values. The higher relative AOD values (per unit of body weight) in female rather than male swimmers might be explained by the sex-specific LBM content, since LBM has shown an inverse effect on all estimates for the assessment of AOD (i.e. V̇O_2demand_, V̇O_2Ac_, and slope). Indeed, the meaning of the negative observed correlation between the relative AOD and LBM is that the level of anaerobic activation is higher among those with low LBM, in order to match a given percentage of energy contribution with anaerobic sources, considering that there is no difference in total energy demand (%V̇O_2peak_) and level of performance (%vV̇O_2peak_) during each trial, and sexes did not differ with regard to V̇O_2peak_ relative to body weight. Moreover, the negative observed correlation between the relative V̇O_2Ac_ and LBM is evidencing the increased requirement of V̇O_2_ among female (unlike male) swimmers during each performance, which further contributed to increase the percentage of AOD to total VO_2_ demand for each trial. Although LBM plays a role in distinguishing the AOD response between sexes, future studies should better understand the influence of body composition on anaerobic conditioning after training planned to increase regional and total lean mass, and hence, also addressing whether these adjustments on energetic profile and muscle mass might account to supramaximal exercise tolerance and time-performance improvements.

## Supplementary Information

**Additional file 1**. Preliminar overview of dataset.

## Data Availability

The datasets used and/or analysed during the current study are available from the corresponding author on reasonable request. Preliminar overview of dataset can be see in Supplementary information (Additional file 1).

## Abbreviations

AOD: Accumulated oxygen deficit
LBM: Lean body mass
O_2_: Oxygen
V̇O_2max_: Maximal oxygen uptake
iV̇O_2max_: Exercise intensity corresponding to V̇O_2max_
V̇O_2Ac_: Accumulated oxygen uptake
V̇O_2demand_: Predicted O_2_ demand
V̇O_2_: Oxygen uptake
V̇O_2peak_: Peak oxygen uptake
vV̇O_2peak_: Velocity at peak oxygen uptake
